# Surface Functionalization of 2D MXenes: Trends in Distribution, Composition, and Electronic Properties

**DOI:** 10.1021/acs.jpclett.0c03710

**Published:** 2021-03-03

**Authors:** Rina Ibragimova, Paul Erhart, Patrick Rinke, Hannu-Pekka Komsa

**Affiliations:** † Department of Applied Physics, Aalto University, P.O. Box 11100, 00076 Aalto, Finland; ‡ Department of Physics, Chalmers University of Technology, S-412 96 Gothenburg, Sweden; ¶ Microelectronics Research Unit, University of Oulu, P.O. Box 8000, 90014 Oulu, Finland

## Abstract

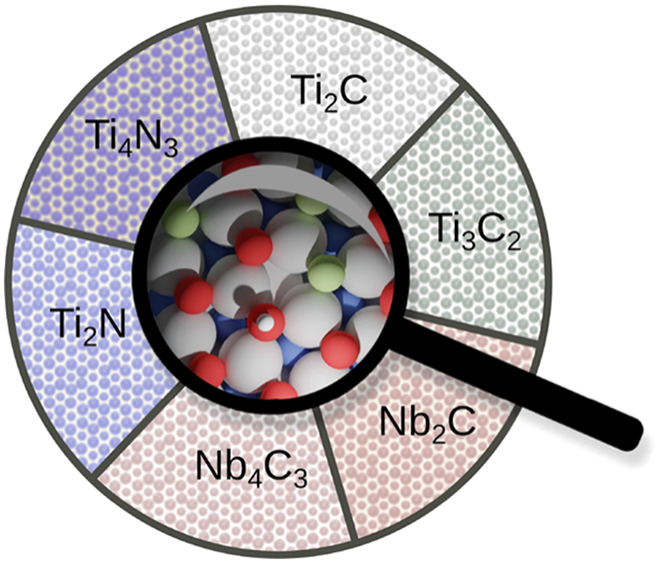

Using a multiscale computational scheme, we study the trends in distribution and composition of the surface functional groups −O, −OH, and −F on two-dimensional (2D) transition metal carbides and nitrides (MXenes). We consider Ti_2_N, Ti_4_N_3_, Nb_2_C, Nb_4_C_3_, Ti_2_C, and Ti_3_C_2_ to explore MXenes with different chemistry and different number of atomic layers. Using a combination of cluster expansion, Monte Carlo, and density functional theory methods, we study the distribution and composition of functional groups at experimentally relevant conditions. We show that mixtures of functional groups are favorable on all studied MXene surfaces. The distribution of functional groups appears to be largely independent of the type of metal, carbon, or nitrogen species and/or number of atomic layers in the MXene. We further show that some properties (e.g., the work function) strongly depend on the surface composition, while others, for example, the electric conductivity, exhibit only a weak dependence.

MXenes are 2D materials with the general composition M_*n*+1_X_*n*_, where M is a transition metal and X is carbon or nitrogen.^1,2^ MXenes include materials with different M and X combinations,^1−4^ ordered materials with different metal combinations in outer and inner layer M′_2_M″X_2_,^5^ and phases with ordered divacancies in the structure M_1.33_X called i-MXenes.^6^ Herein, we focus on MXenes with one M element in the structure, the combination of element M and X and different numbers of atomic layers: M_2_X,^1,2^ M_3_X_2_, M_4_X_3_,^7,8^ and M_5_X_4_.^9^ They possess extraordinary electronic, mechanical, optical, thermal, and catalytic properties.^10−17^ During wet-etching synthesis of MXenes,^1,4^ their surfaces adsorb functional groups such as −O, −OH, and −F.^18−24^ Even though a variety of MXene properties such as work function, hydrophilic behavior, and catalytic activity are ascribed to the surface functionalization,^1,25−27^ the structure and composition of the functionalized surfaces remain unknown for most MXenes.

To date, several experimental studies have reported significant variations in the surface composition of freshly prepared MXenes.^7,28−37^ However, the experimental characterization of MXene surfaces is challenging because (i) the surface contains light elements such as H, O, and F; (ii) the surface is often contaminated with water and precursors remaining after etching, and (iii) variations in the experimental conditions aggravate systematic studies. For example, the XPS analysis of Halim et al.^7^ reveals that Ti_3_C_2_, Ti_2_C, Ti_3_CN, Nb_2_C, and Nb_4_C_3_ all exhibit a mixture of O/OH/F when etched with HF, although with slightly different compositions. Conversely, Ti_2_N and Ti_4_N_3_ obtained from molten-salt etching^28−30^ accommodate only a mixture of O and OH, and no F. It is not known whether the absence of F is triggered by the material itself or is a result of the synthesis method. To date, no clear picture has emerged on what factors determine the composition and distribution of surface functional groups.

First-principles calculations complement experimental studies and provide atomistic insight into the surface functionalization. However, to date the majority of theoretical studies considered only pure terminations of O, F, or OH,^1,25,38−55^ and there are only a few studies involving mixtures of functional groups.^56−58^ To address mixed functionalization, we recently developed a multiscale computational scheme to find the equilibrium composition of statistically averaged distributions of −O, −OH, and −F functional groups on MXene surfaces, also taking into account the interactions with a solvent.^59^ The scheme was employed to study Ti-based carbides for certain experimental conditions characterized by the pH value, the open-circuit potential (OCP), and the growth temperature. However, a systematic study of surface functionalization of MXenes is still missing.

In this Letter, we remedy this situation and provide a systematic, atomic-scale analysis of surface functionalization for titanium (Ti) and niobium (Nb) carbides and nitrides of varying thickness. The diversity of MXenes provides a large design space, which we narrow down here by exploring three of its dimensions: the type of metal (M), the type of X element, and the number of atomic layers *n*. Moreover, we select materials that have already been synthesized to be able to compare to experimental data. Furthermore, we exclude elements that give rise to magnetic properties and/or where strong correlations may be expected. On the basis of these criteria, we have selected the following MXenes: Ti_2_N, Ti_4_N_3_, Nb_2_C, Nb_4_C_3_, Ti_2_C, and Ti_3_C_2_ for our study (Figure 1a).

**Figure 1:**
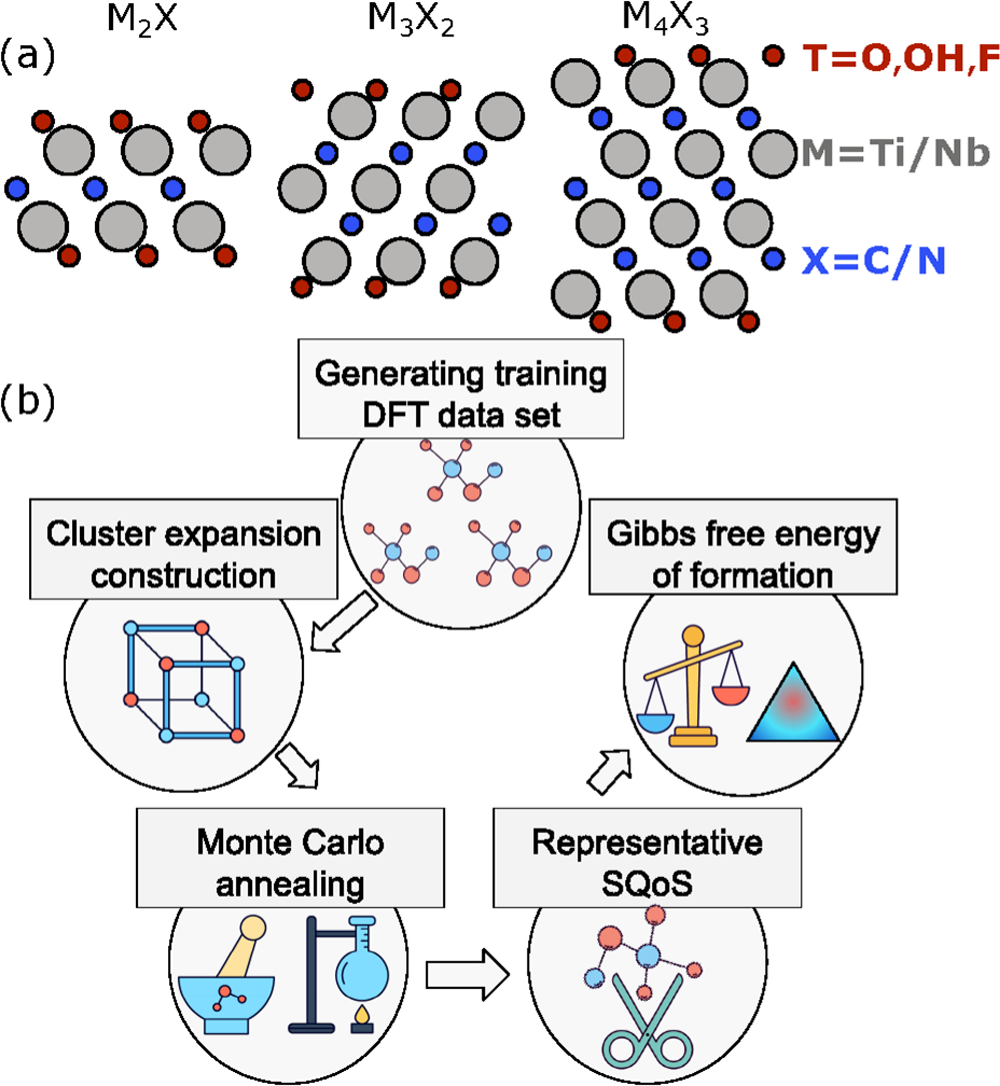
(a) Side-view structures of the considered MXenes of different thickness: M_2_X, M_3_X_2_, and M_4_X_3_. (b) Schematic of our multiscale computational scheme.

Our multiscale approach is schematically depicted in Figure 1b. First, we generate a set of three-component (F, O, and OH) reference structures for each considered MXene. For these structures, we perform density functional theory (DFT) calculations using the VASP code.^60^ Next, we construct a cluster expansion (CE) Hamiltonian for each MXene using the icet code.^61^ The CE is fitted to the DFT energies for the reference structures using Bayesian linear regression via the automatic relevance detection (ARD) scheme^62^ (see fit quality in Figure S1b). We include pair clusters up to the fourth-nearest neighbors and triplet clusters up to first-nearest neighbors in the CE (Figures S1a and S2). We then use Monte Carlo (MC) simulations to sample the configurational space and to compute the configurational free energies for different surface terminations (see the Supporting Information for simulation details).^63^ In this way, we obtain the equilibrium distribution of functional groups on different MXenes. To further analyze the properties of thermodynamically averaged structures, we use the special-quasiordered-structures method (SQoS)^64,65^ and generate representative 4 × 4 × 1 supercells for 12 fixed concentrations of functional groups. The generated structures exhibit a distribution of functional groups that closely mimic those observed in the larger supercells sampled during the MC simulations. Finally, we calculate the Gibbs free energy of formation for the generated structures in solution over the whole range of composition of the functional groups, carefully accounting for the role of experimental factors, such as temperature, pH, and open-circuit potential.

*Distribution of Functional Groups*. We start with the analysis of functional group distributions obtained from the MC simulations, which were carried out for 12 structures with compositions M_*n*+1_X_*n*_–(O_*x*_OH_*z*_F_1–*x*–*z*_)_2_, where *x* and *z* vary from 0 to 1 in steps of 0.25. Radial distribution functions for O–OH pairs of all MXenes at (O_0.5_OH_0.5_)_2_ composition are shown in Figure 2a. The radial distribution functions are almost identical for all six systems, which indicates that the distribution of functional groups does not depend on the type of MXene. Therefore, in the following we show only the distributions for Ti_2_N.

**Figure 2:**
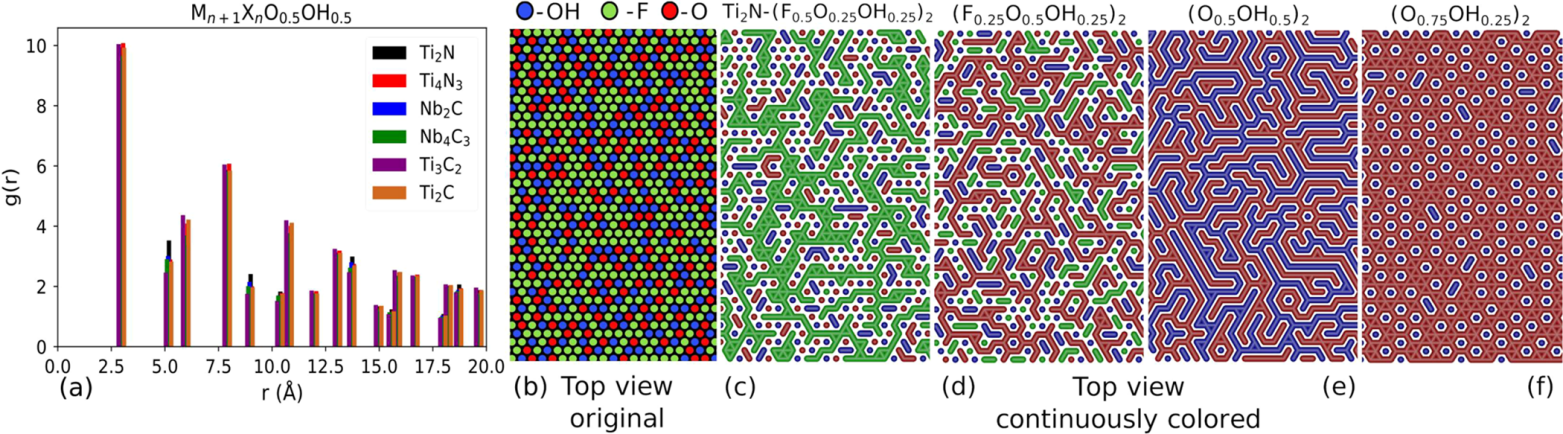
(a) O–OH radial distribution function for all systems and surface structures of (b) Ti_2_N(F_0.5_O_0.25_OH_0.25_)_2_ (original schematics of surface coverage), (c) Ti_2_N(F_0.5_O_0.25_OH_0.25_)_2_, (d) Ti_2_N(F_0.25_O_0.5_OH_0.25_)_2_, (e) Ti_2_N(O_0.5_OH_0.5_)_2_, (f) Ti_2_N(O_0.75_OH_0.25_)_2_. In panels c–f the nearest neighbors of the same type are connected to highlight the ordering.

The surface distribution for excess F (Ti_2_N–(F_0.5_O_0.25_OH_0.25_)_2_ in Figure 2b,c) shows that fluorine atoms are arranged in strips with a thickness of one or two atomic rows. The spaces between F-strips are mainly filled with an ordered distribution of alternating O and OH groups. Likewise, excess O [(F_0.25_O_0.5_OH_0.25_)_2_ in Figure 2d] leads to the formation of oxygen-containing strips. F and OH alternate in the remaining space, although with more F–F and OH–OH pairs than for excess F. The binary structure with 50% of O and OH is depicted in Figure 2e and exhibits alternating O and OH strips. In the case of (O_0.75_OH_0.25_)_2_ in Figure 2f, the OH groups are evenly distributed within the O groups.

The strip patterns were observed for all studied systems independent of the composition in terms of metal species (M) and carbon or nitrogen (X) as well as the number of atomic layers (*n*). Overall, the functional groups clearly mix and do not exhibit phase separation or agglomeration. The mixing happens on the atomic scale; that is, the connected features are atomically thin. We attribute the emergence of strip patterns to the triangular lattice of MXenes. In O–OH binaries, maximizing the number of O–OH bonds leads to a strong geometrical frustration in all MXene systems that usually manifests itself in the formation of strip patterns as observed here. The geometrical frustration usually gives rise to a manifold of ground states rather than a single stable ground state.^66^ As a result, the system will be sensitive to slight perturbations, meaning that slight variations of the external conditions can easily lead to changes in the surface group distribution.

The ternary diagrams of the mixing energy are depicted in Figure 3, which shows the mixing energy as a function of the concentrations of −F, −O, and −OH functional groups for the considered MXenes. We observe a pronounced minimum for binary compositions with 50% O and 50% OH (O_0.5_OH_0.5_) for all systems. The absolute mixing energies, however, vary with no clearly discernible trend. Nb_4_C_3_ has the smallest mixing energy of −0.13 eV and Ti_3_C_2_ the largest (− 0.2 eV). With an increasing number of atomic layers, the absolute values of the mixing energy decrease for Ti_4_N_3_ and Nb_4_C_3_ but not for Ti_3_C_2_.

**Figure 3:**
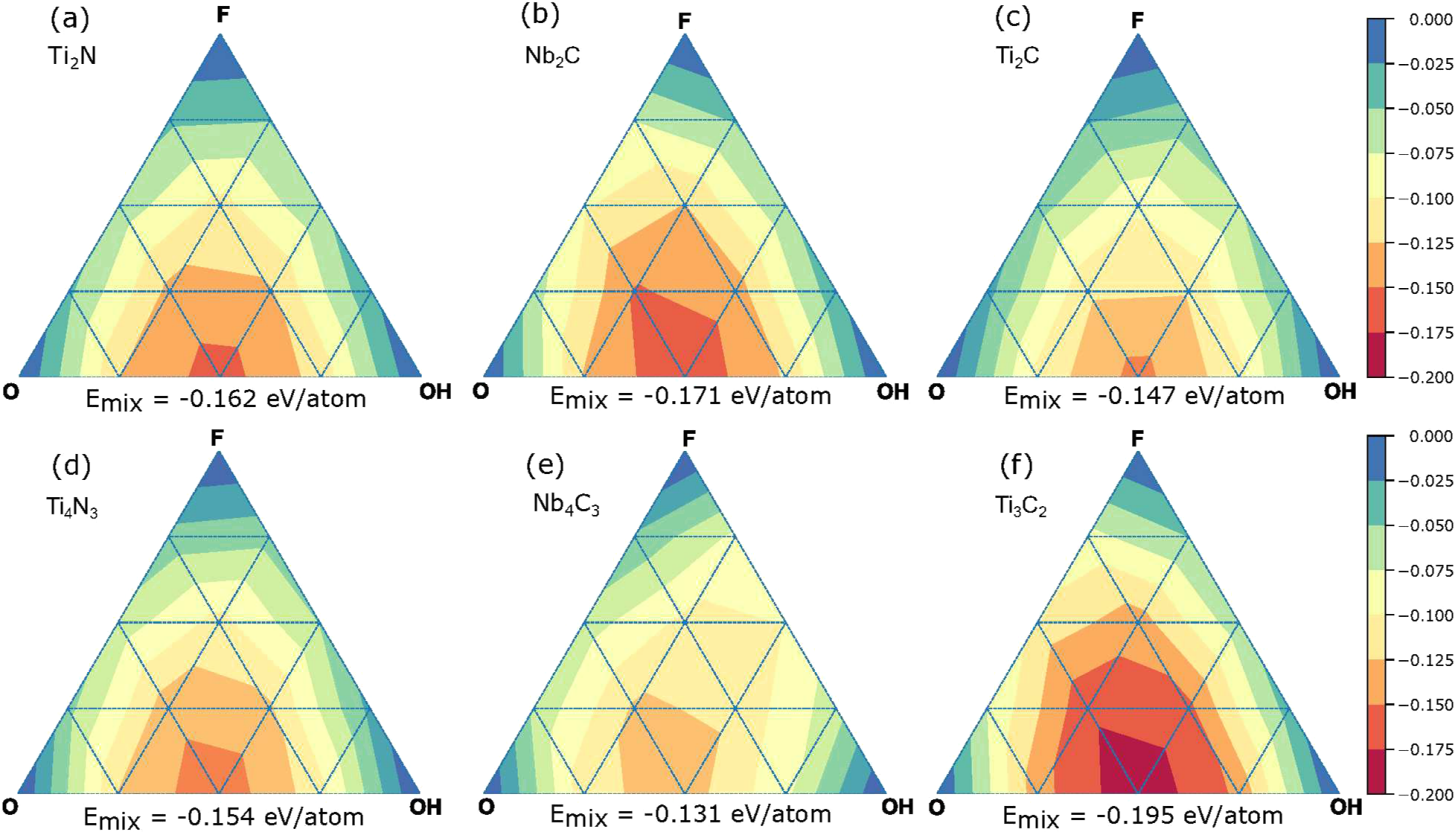
Mixing energy (in eV per MXene unit cell; each unit cell contains two surface sites) of (a) Ti_2_N, (b) Nb_2_C, (c) Ti_2_C, (d) Ti_4_N_3_, (e) Nb_4_C_3_, and (f) Ti_3_C_2_ as a function of the concentrations of −O, −F, and −OH.

The strong preference toward mixing in our CE indicates that the surface functionalization is primarily determined by interactions between functional groups. While changes in the Fermi-level position could change the bond strength between functional groups and the substrate, this would lead to similar mixing energies for all structural configurations with the same composition, clearly contradicting our CE results. Moreover, if the bonding energy depended on the Fermi-level position, the mixing energy curves were unlikely to look so similar because in different MXenes the Fermi level falls into different regions of the metal d-band. The similarity of the mixing energy diagrams (Figure 3) points toward interactions that are inherent to the functional groups. Because the distance between the functional groups is too large for direct chemical bonding, the functional group distribution is likely dictated by electrostatic interactions. In the case of differently charged functional groups, the electrostatic energy of a mixed system is always lower than for a segregated system. To this end, we evaluated the charges associated with each atom, using the Bader method.^67^ The average number of electrons that are associated with O and OH increases almost linearly with the amount of OH in the system (Figure S4a,c). This dependence is similar in every studied system, and we found no clear correlation with the maximum mixing energy or substrate chemistry and the number of atomic layers. The charge difference between O and OH groups is similar (0.6–0.7 e) in all materials, consistent with the similarity of the distributions of functional groups and the mixing energy diagrams. On the other hand, the charges of N and C atoms do depend on the surrounding metallic species, which subsequently changes the charge in the metal atom and thus the filling of the metal d-band, as will be seen from the density of states below.

*Equilibrium Composition*. Next we evaluate the thermodynamic equilibrium composition of surface terminations in HF solution after the etching by means of Gibbs free energy of formation calculations. We assume that all species are in equilibrium in order to set the chemical potentials for F, O, and H and link them to the pH of the solution as well as to the open-circuit potential and the temperature. Full details of our approach can be found in ref (59).

Figure 4 shows the ternary diagrams of the Gibbs free energy of formation for mixed surface terminations. The chemical potentials are determined at standard hydrogen electrode (SHE) conditions, where the pH is equal to 0, and the electron chemical potential is fixed to the calculated *U*_SHE_ = 4.7 eV (see the Supporting Information for calculation details). SHE conditions correspond to H_2_ molecules splitting into two H^+^ ions, and we anticipate that the functionalization happens near those conditions because of the high H^+^ content in the solution. For Ti-based nitrides (Figure 4a,d), we find a minimum in the Gibbs free energy at O_0.75_OH_0.25_ composition. The same composition of O_0.75_OH_0.25_ was found for Nb-based carbides (Figure 4b,e). However, for the Ti-based carbides (Figure 4c,f), the minimum occurs at O_0.50_OH_0.25_F_0.25_. In all systems, the number of atomic layers does not affect the equilibrium composition. The difference in energy between fully O-terminated and O_0.75_OH_0.25_ configurations (Ti nitrides and Nb carbides) is rather small (0.02–0.05 eV), indicating that both phases, O and O_0.75_OH_0.25_, might be accessible during synthesis. We note that the minima of mixing energies were always found at the same composition (Figure 3) for all systems; however, the Gibbs free energy minima are located at different compositions. This difference is caused by the different values of the formation energies of the pure functionalized surfaces. For example, for Nb-based MXenes, a strong preference for the O-terminated surfaces seems to overcome the mixing energy gain.

**Figure 4:**
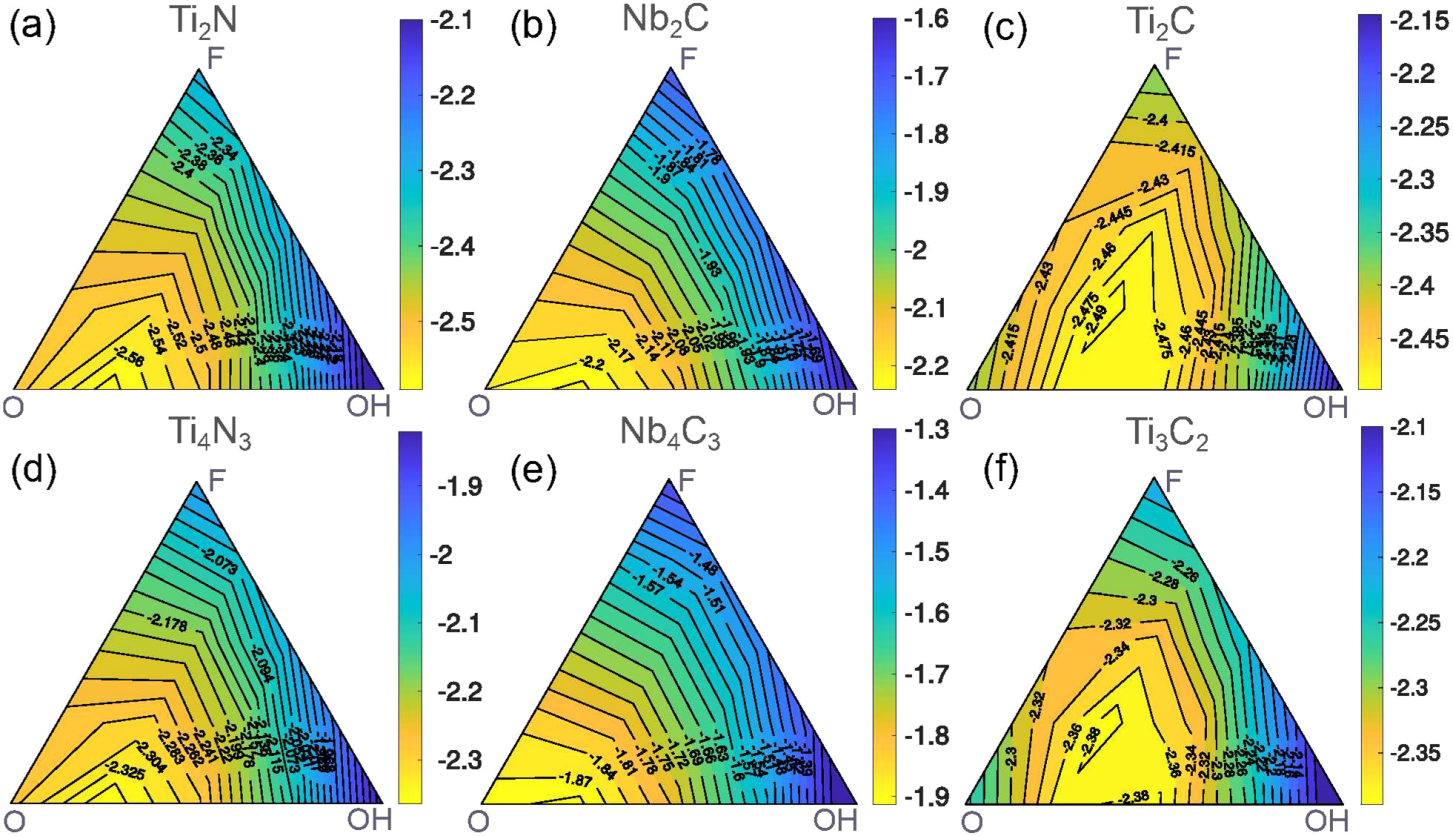
Gibbs free energy of formation for (a) Ti_2_N, (b) Nb_2_C, (c) Ti_2_C, (d) Ti_4_N_3_, (e) Nb_4_C_3_, and (f) Ti_3_C_2_. The diagrams are plotted for SHE conditions (pH = 0; *U* – *U*_SHE_ = 0 V).

Figure 5a depicts the surface composition of Nb_2_C as a function of OCP and pH. We also show full composition diagrams for all systems with different M and X in Figure 5b, where we fixed the pH value to 0 for the variation of the potential and the value of the potential to 0 eV for the pH variation. Surfaces of Ti-based nitrides have a high O content (75%) with 25% OH in the whole pH range. Increasing the potential to 0.2 eV removes the OH groups and leads to a fully O-terminated surface. For Nb-based carbides, the situation is similar, only that the transition to a fully O-covered surface also happens for pH variations. Ti-based carbides exhibit the most complex behavior. Upon increasing the potential, the OH content can be reduced from 50% to 0%. At slightly positive potentials also F can be stabilized in the mix. A similar switch from an O–OH mixture to a F-containing one is observed for pH variations around a pH value of 2.0. While an increase in pH can be problematic for etching systems with strong bonds, the applied electrode potential can be tuned^68^ and thus offers a potentially easy way to affect the surface composition.

**Figure 5:**
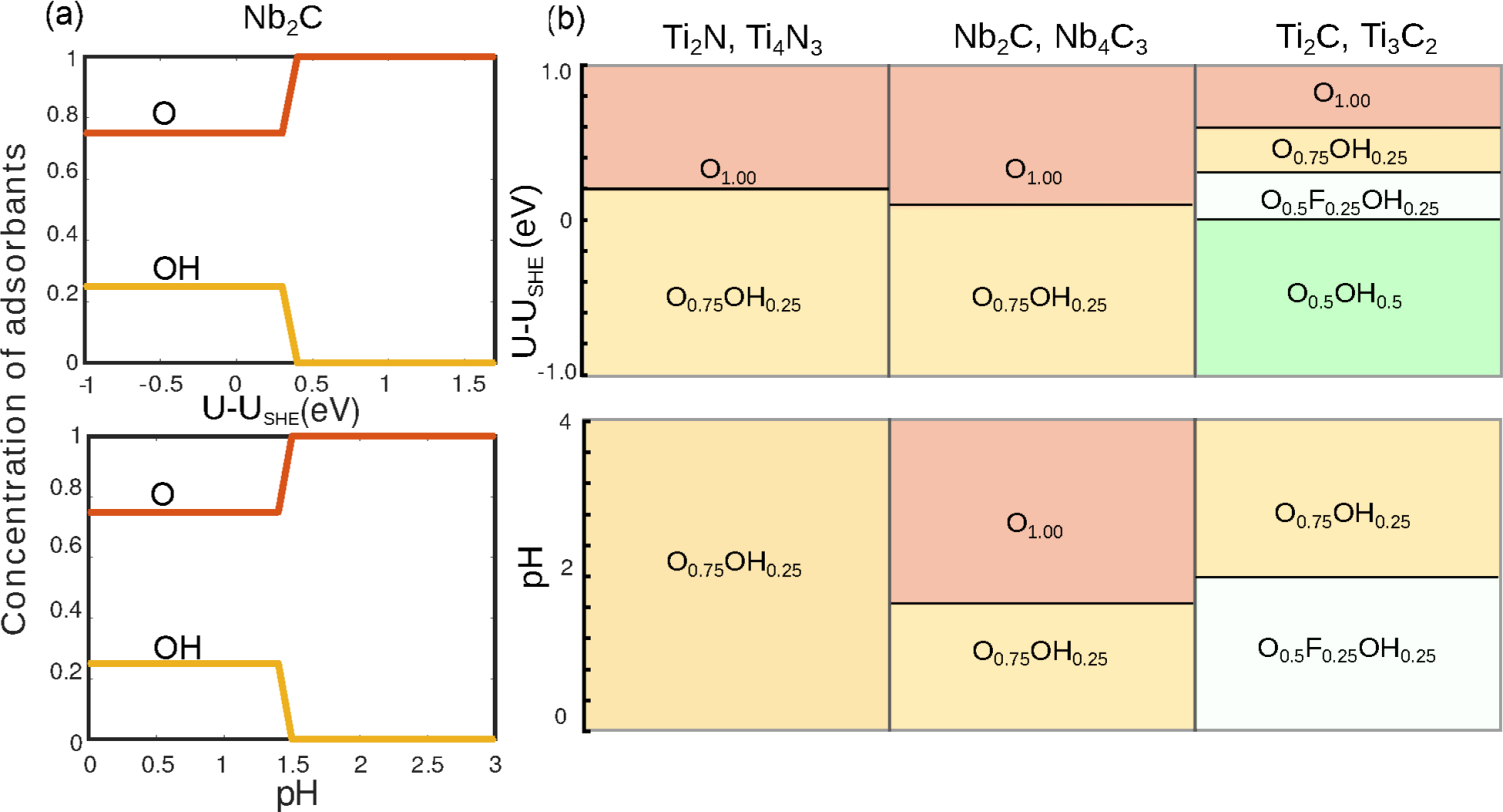
(a) Composition with the lowest energy for Nb_2_C dependent on the open-circuit potential (top panel) and the pH (bottom panel). (b) Summary of stable compositions for all systems as a function of the open-circuit potential (at pH 0; top panel) and pH (at *U* – *U*_SHE_ = 0 V; bottom panel).

Comparing our surface phase diagrams depicted in Figure 5 to experiment, we first note that experimental observations on the surface composition show significant scatter.^7,28−37^ Ti-based carbides are the most studied systems thus far, and experimental findings could be cherry-picked to support almost any computational result.^20,27,36,69,70^ Focusing only on the high accuracy measurement techniques such as neutron scattering and nuclear magnetic resonance spectroscopy, it emerges that Ti-based carbides have the most mixed compositions out of all considered structures.^36,70^ In ref. (71), the pH of the solution was varied from −0.9 to 1.4 by keeping the concentration of HF fixed while changing the other components in the solution. However, the resulting O, OH, and F compositions were similar in all cases. Both findings are in general agreement with our calculated results.

Etched Ti_2_N surfaces (in a mixture of potassium fluoride and hydrochloric acid) accommodate all three functional groups albeit with a predominance for O.^35^ Conversely, molten-salt synthesis functionalizes the surfaces of Ti_2_N and Ti_4_N_3_ mostly with O and OH,^29,30^ despite the presence of F during etching. While neither of these experiments directly corresponds to the conditions in our simulations, they tend to agree with our results so far that smaller F concentrations are observed. Contradicting experimental observations are reported for HF-etched Nb-based carbides. Mixtures of −OH and −F functional groups were detected in Nb-based MXenes in nuclear magnetic resonance (NMR) spectroscopy,^72^ while another study reported high O concentrations for HF-etched Nb_2_C and nonstochiometric mixtures of functional groups for Nb_4_C_3_.^7^ In all of these materials, OH appears to be the minority species, which is in agreement with our results. The presence of F in the case of Ti nitrides and Nb carbides (and lack thereof in our calculations) can arise from kinetic aspects or too low F chemical potentials in our calculations, whereas in experiments the F concentration may be higher because the concentration of available species between the sheets may differ from that of the solution.

*Composition-Dependent Properties*. Next we analyze how the properties of MXenes change with surface functionalization and its composition. For example, previous computational and experimental studies found that the work function of MXenes strongly depends on the composition of the functional groups.^1,26,27,38,58,59^ This dependence was utilized to engineer the work function and band alignment in solar cells by adding MXene layers to perovskites.^73^

We have calculated the work functions over the whole range of −O, −OH, and −F concentrations for all the considered systems (Figure S5). The work function depends linearly on the concentration of O, F, and OH. Fully O- and F-terminated structures have the highest work functions above 5 eV, and the values decrease with an increase of OH content to about 2 eV. The results for pure terminations agree with earlier computational results,^8,38^ while the values for the whole range of O, OH, and F concentrations are reported here for the first time. Previous calculations found that the work functions are governed by the dipoles formed in the functionaliztion layer.^38^ Consistent with that notion, we observe only a small variation of the work function values for different MXenes. The work function of MXenes therefore depends primarily on the functional group composition and little on the M or X species.

MXenes are attractive materials because of their high electrical conductivity. For a first assessment of the conductivity, we here inspect the electronic density of states (DOS). We showed previously that the DOS at the Fermi level of Ti-based carbides only weakly depends on the accessible functional group composition.^59^ In Figure 6 we show the total and atom-projected DOSs for Ti_2_N and Nb_2_C for different O and OH concentrations. The DOSs for other calculated systems are shown in Figure S6. F groups are expected to behave similarly to OH groups, because they both accept one electron. Similar to Ti-based carbides, the Fermi level falls inside the metal d-band independent of functionalization, and the same is true for the studied systems regardless of the type of M and X species (with the exception of fully O-terminated Ti_2_C, which becomes semiconducting). Assuming the average carrier relaxation time is insensitive to surface composition, this suggests that the electrical conductivity is only weakly affected by functionalization.

**Figure 6:**
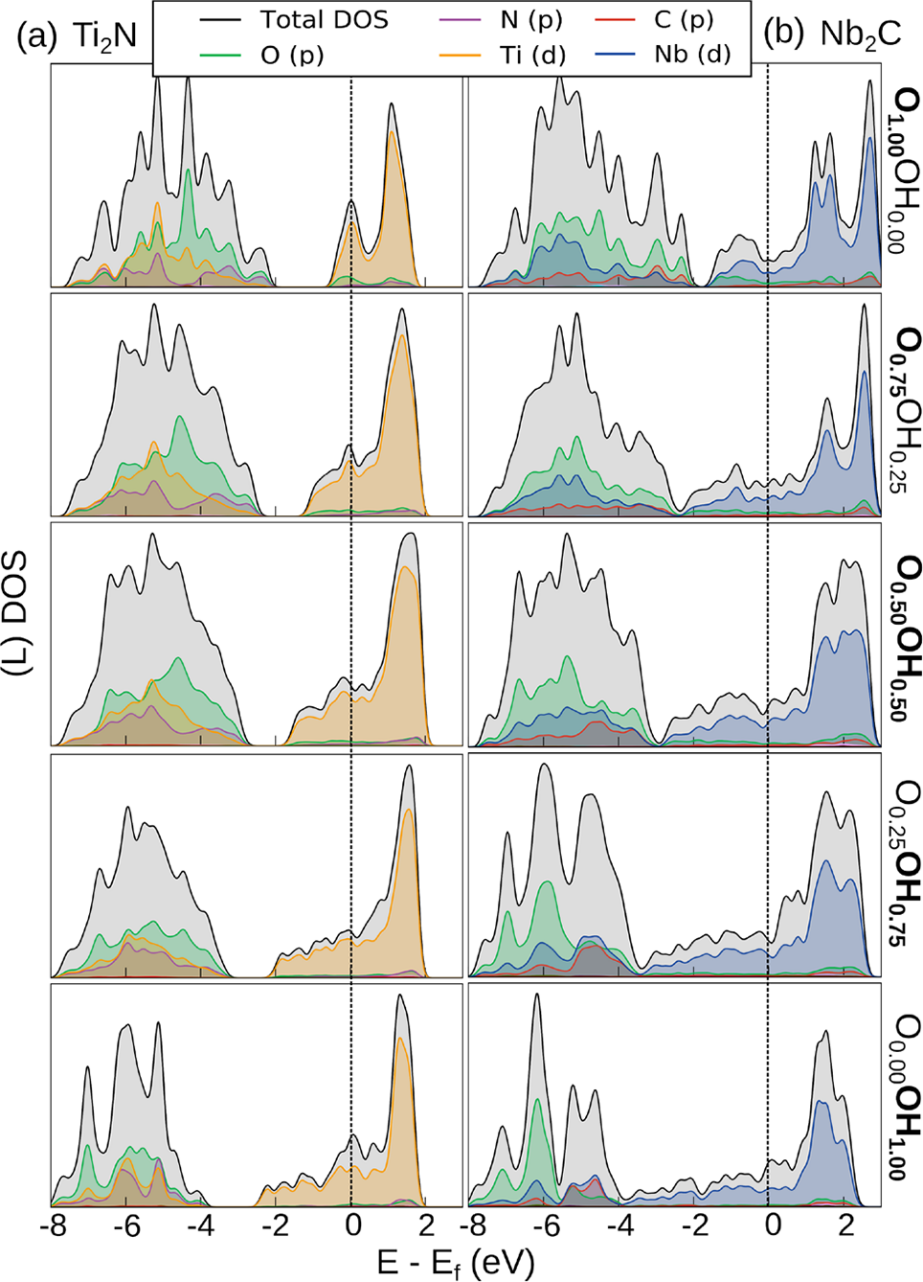
Atom-projected density of states for (a) Ti_2_N and (b) Nb_2_C SQoSs with different O and OH composition. The top panel corresponds to the fully O-terminated surface, and the OH content gradually increases toward the bottom panel. The vertical dashed lines indicate the Fermi level position.

Conversely, the DOS at the Fermi level increases concomitantly with the number of layers (Figure S6). Furthermore, for Nb-based systems the Fermi level is located at a higher DOS region than for any of the other calculated MXenes as a result of the higher number of valence electrons. Because the metallic conductivity of these MXenes is carried via the transition metal d-band states at Fermi level and the Fermi level remains within the d-band for most accessible surface compositions, the conductivity is weakly affected by the composition of the adsorbate layer.

In conclusion, we have systematically studied surface functionalization of six 2D MXenes M_*n*+1_X_*n*_ by −O, −F, and −OH. We explored the chemical space of MXenes considering different metallic species (M = Ti, Nb), a variation of X species (X = C, N), and variation in the number of atomic layers (*n* = 2, 3, 4). Using a multiscale computational scheme, we calculated the distribution of functional groups and their mixing energies. The surface functionalization of different MXenes exhibits similar distributions and mixing energies, which we propose are governed by interactions between −O, −F, and −OH and the geometry of the triangular lattice rather than the chemical nature of the M and X species or the number of atomic layers (*n*). To simulate realistic synthesis conditions, Gibbs free energy of formation diagrams for different compositions of functional groups depending on pH and the potential were constructed. The Gibbs free energy of formation indicates a prevalence of O functionalization, regardless of M, X, and *n* variation, and suggests that functionalization of the surface is driven by external conditions and not the type or thickness of the MXene. Finally, the work function of the surface varies dramatically, and linearly, with functional group composition, whereas the DOS at the Fermi level is only weakly affected by the surface groups.

MXenes are a wide class of materials with various properties and structures, but for most MXenes the functional group distribution has not been studied experimentally. While we focused on only a few of the most common MXenes, the surface functionalization behavior can be different for MXenes with different structures and properties than those of the systems studied in this work. Nevertheless, we hope that our findings will also be helpful in understanding the structure and properties of other MXenes.

## Supporting Information Available

Methodology and results (PDF)
