# Supplementary material for: Surface Functionalization of 2D MXenes: Trends in Distribution, Composition, and Electronic Properties
Source: J Phys Chem Lett. 2021 Mar 3;12(9):2377–84. doi: 10.1021/acs.jpclett.0c03710 (PMC8041312; doi:10.1021/acs.jpclett.0c03710)
Supplement: Supplementary file 1 — jz0c03710_si_001.pdf [file jz0c03710_si_001.pdf]

Supplementary Information for "Surface functionalization of 2D MXenes: Trends in distribution, composition, and electronic properties"

Rina Ibragimova[1], Paul Erhart[2], Patrick Rinke[1], and Hannu-Pekka Komsa[1,3,*]

[1] Department of Applied Physics, Aalto University, P.O. Box 11100, 00076 Aalto, Finland
[2] Department of Physics, Chalmers University of Technology, S-412 96 Gothenburg, Sweden
[3] Microelectronics Research Unit, University of Oulu, P.O. Box 8000, 90014 Oulu, Finland
[*]hannu-pekka.komsa@oulu.fi

1 Methodology

1.1 Cluster Expansion

We start by generating a cluster expansion, which is constructed based on the assumption that energy of the system can be expanded on a series of clusters (or nearest neighbours depicted on Fig. S1 (a)) and their effective interactions (ECI) Fig. S2. The model Hamiltonian is then obtained by fitting ECI to the set of energies calculated by DFT method. For each system, we produce a DFT set of structures with binaries and ternaries of functional groups within a full range of O, OH, and F concentrations. We used set of 140 structures for Ti_2N, 80 structures for Ti_4N_3, 71 structures for Ti_2C, 106 structures for Ti_3C_2, 132 structures for Nb_2C, and 119 structures for Nb_4C_3. To evaluate the accuracy of each cluster expansion, we used cross validation scores. Cross validation scores were obtained within a range of 7-15 meV which gives rise to an error at maximum of 10 %. As an example, the scattered plot of DFT energies vs. CE energies as a function of O concentration is shown for Ti_2N and Nb_2C in Fig. S1(b). In both regressions, the predicted energies are reproducing the actual calculated energies well, with no outliers. In a low mixing energy region, the structures with no presence of O are mostly common, whereas in a higher mixing energy region, mostly structures with sufficient amount of O are present.

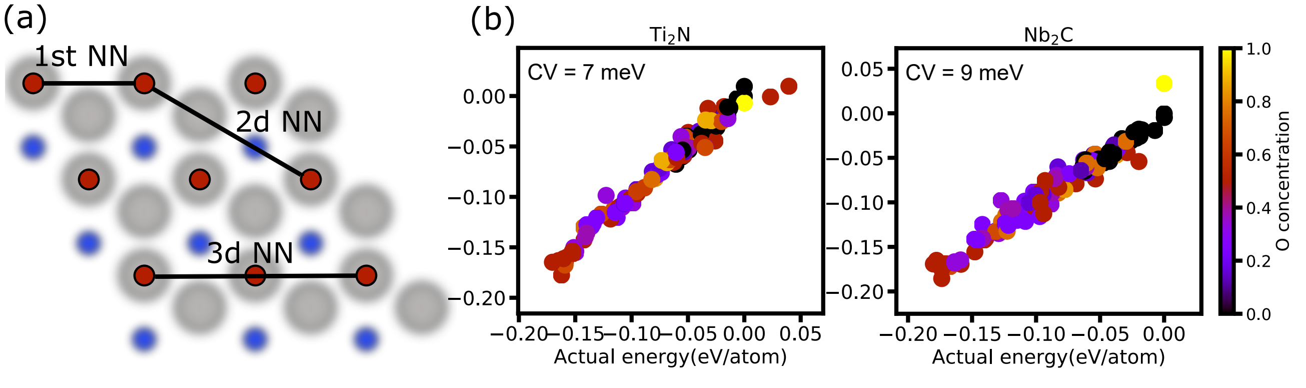

Figure S1: (a) Atomic structure of M_2XT_2 in top view with clusters used in the expansion. (b) Predicted CE energy vs. DFT energy as a function of O concentration for Ti_2N and Nb_2C.

1.2 Monte Carlo simulations and special quasi-ordered structures

For MC sampling we used a 40x40x1 conventional supercell with 3200 functional group sites. Sampling was carried out using canonical ensemble at the temperature decreased from 2000 K to 300 K with a 100 K intervals. The calculations are carried out with 200000 steps, where the number of steps was tested with respect to the convergence of energy.

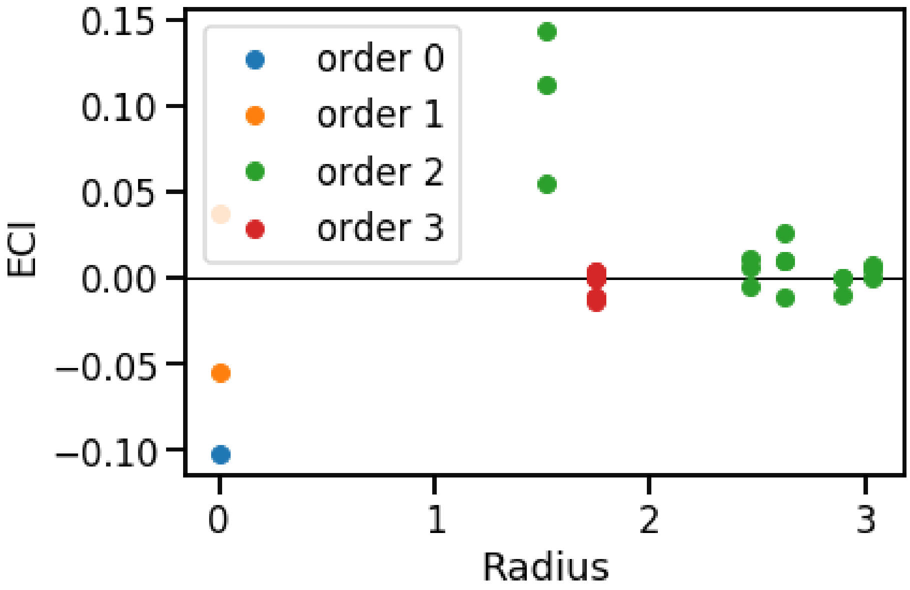

Figure S2: Effective cluster interactions for Ti_2N as a function of clusters radius.

Best representative structures were acquired using a special quasi-ordered structures method (SQoS)[10, 5]. Cluster correlations from larger equilibrated structures were used to generate smaller representative structures by minimizing the objective function. Minimization is done by canonical Monte Carlo simulation with 100000 steps for the 4x4x1 supercells. The calculated structures are mimicking distribution of functional groups of the larger structures.

1.3 Free energy calculations

In order to compare the stability of MXene sheets with different surface terminations, we determine Gibbs free energy of formation, which is obtained by finding Gibbs free energy for all the constituents. Here we define it for the terminated sheet MXT with respect to the bare, unterminated sheet MX as:

$$\Delta_f G^\circ(\mathrm{MXT}) = G^\circ(\mathrm{MXT}) - G^\circ(\mathrm{MX}) - \sum_i n_i \widetilde{\mu}_i \tag{S1}$$

where n_i are the number of termination atoms of type i and $\widetilde{\mu}_i$ are their chemical potentials. G is the Gibbs free energy of the system and $^\circ$ refers to standard conditions: room temperature and in solution with pressure $p = 1$ atm. Consequently, the free energy of the sheet should include the vibrational contributions as well as the interaction with the solution. We assume that the two contributions do not depend on each other and thus

$$G^\circ(\mathrm{MXT}) = E(\mathrm{MXT}) + \Delta_{\mathrm{vib}} F(\mathrm{MXT}) + \Delta_{\mathrm{sol}} E(\mathrm{MXT}) \tag{S2}$$

where $E(\mathrm{MXT})$ is the DFT total energy, $\Delta_{\mathrm{vib}} F$ is phonon contribution to free energy at room temperature evaluated in vacuum, and $\Delta_{\mathrm{sol}} E$ is the solvation energy evaluated at $T = 0$ using implicit solvation models. (pV term can be ignored at $p = 1$ atm.) We use vibrational and solvent contributions for calculating formation energies, since the order of magnitude is comparable, we use the values from calculated earlier Ti_2C and Ti_3C_2 [3].

Note, that following framework is adopted from the theoretical concepts proposed by Todorova and Neugebauer [9]. We determine the chemical potentials of H, O, and F in accordance to the experimental conditions. Chemical potential of O is determined via chemical potential of H and water: $\mu(O) = \mu(H_2O) - 2\mu(H)$, where $\mu(H_2O)$ is solvated water. We use the experimental Gibbs free energy of formation

$$\widetilde{\mu}^\circ(H_2O) = \widetilde{\mu}^\circ(O) + 2\widetilde{\mu}^\circ(H) + \Delta_f G(H_2O) \tag{S3}$$

where $\Delta_f G(H_2O) = -237.14$ kJ/mol $= -2.458$ eV [2].

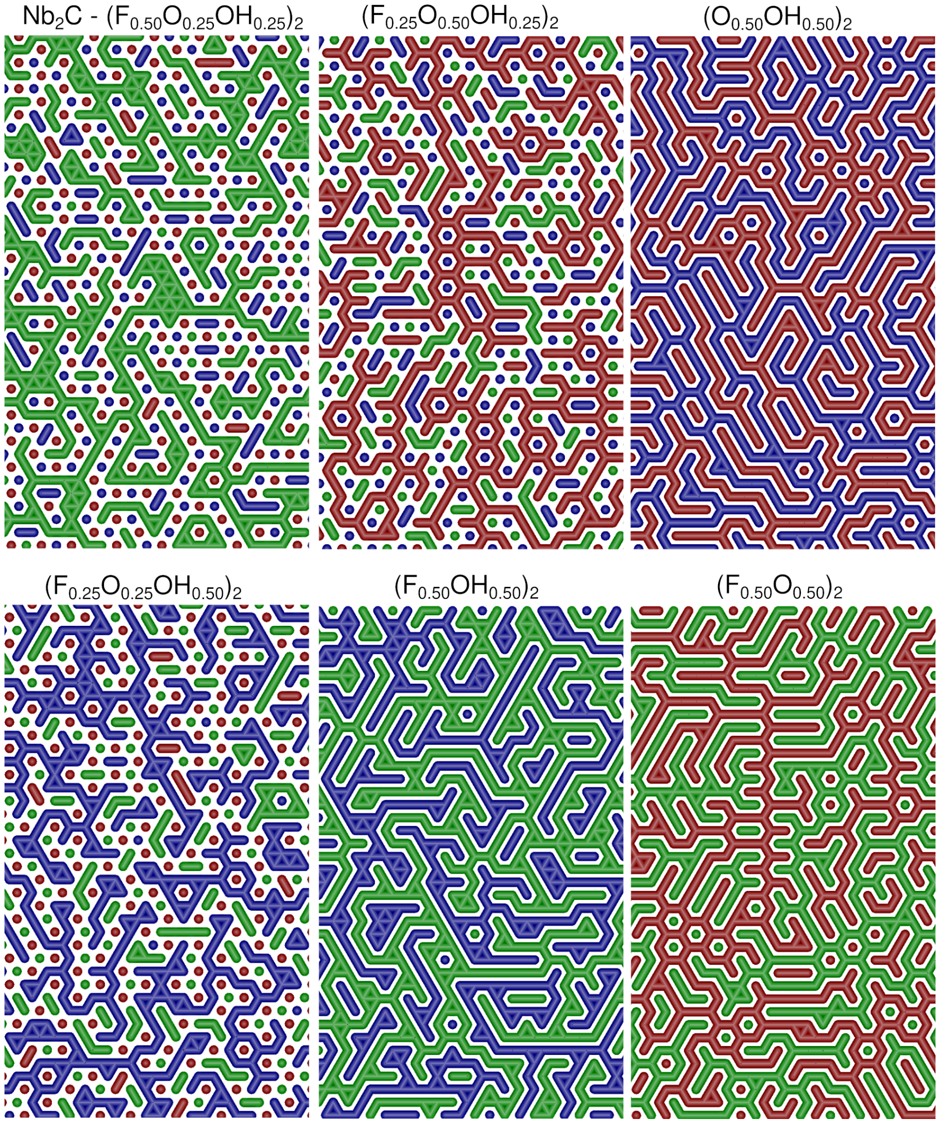

Figure S3: Surface structures of $Nb_2C(F_{0.5}O_{0.25}OH_{0.25})_2$, $Nb_2C(F_{0.25}O_{0.5}OH_{0.25})_2$, $Nb_2C(O_{0.5}OH_{0.5})_2$, $Nb_2C(F_{0.75}O_{0.25}OH_{0.25})_2$, $Nb_2C(F_{0.5}OH_{0.5})_2$, $Nb_2C(F_{0.5}O_{0.5})_2$, where the nearest neighbors of the same type are connected to highlight the ordering.

The chemical potential of H^+ ions, would depend on the electron chemical potential. The formation energies of H^+ and F^- ion are written as

$$\Delta_f G(H^+) = \Delta_f G^\circ(H^+) - \mu(H) + \mu_e \tag{S4}$$

$$\Delta_f G(F^-) = \Delta_f G^\circ(F^-) - \mu(F) - \mu_e \tag{S5}$$

where $\Delta_f G^\circ({}^{+/-})$ are the energy of solvated ions. Data are taken from NIST-JANAF thermochemical tables and the hydration energies from Refs. [6, 7]. pH is directly related to the H^+ concentration in the solution:

$$c_{H+} = c_0 \cdot \exp(-\Delta_f G(H^+)/k_B T) = 10^{-pH} \tag{S6}$$

where $c_0 = 55.55$ mol/l is the concentration of H_2O molecules in water. From this and by using Eq. S4 we obtain

$$\begin{aligned} \Delta_f G(H^+) &= \Delta_f G^\circ(H^+) - \mu(H) + \mu_e \\ &= k_B T \cdot [\ln(55.55) + \ln 10 \cdot \mathrm{pH}] \end{aligned} \tag{S7}$$

From Eq. S7 we get the H chemical potential as

$$\mu(H) = \Delta_f G^\circ(H^+) + \mu_e - k_B T \cdot [\ln(55.55) + \ln 10 \cdot \text{pH}] \tag{S8}$$

Similarly, for F chemical potential, the Eq. S4 can be rewritten.

$$\begin{aligned}\Delta_f G(F^-) &= \Delta_f G^\circ(F^-) - \mu(F) - \mu_e \\ &= k_B T \cdot [\ln(55.55) + \ln 10 \cdot \text{pH}]\end{aligned} \tag{S9}$$

and finally

$$\mu(F) = \Delta_f G^\circ(F^-) - \mu_e - k_B T \cdot [\ln(55.55) + \ln 10 \cdot \text{pH}] \tag{S10}$$

Eventually, we have chemical potentials of all species connected to pH, electron chemical potential μ_e, and temperature T. Experimental conditions suggest that T would be constant since the synthesis happens at room temperature (T=298 K). However, pH and electron chemical potential may vary. From the dependence of H chemical potential on the electron chemical potential, we can obtain a computational standard hydrogen electrode (SHE) potential equal to 4.7 eV (pH=0). Further, to present our results according to experimental conditions, we vary pH and open circuit potential (OPC), where OPC or (U-U_{SHE}) would be a difference between negative electron chemical potential and SHE potential ($-\mu_e - U_{SHE}$). Thus, we can vary μ_e and pH in equations (S7, S10), to calculate Gibbs free energies of the systems and their minimum energy compositions of functional groups.

1.4 DFT calculations

All Density functional theory calculations were performed using the Vienna ab initio simulation package (VASP) [4] together with projector augmented plane wave method (PAW) [1]. Perdew-Burke-Ernzerhof exchange-correlation functional for solids (PBEsol) has been used for all calculations [8], which was selected based on benchmarkings performed in Ref. [3]. The optimal plane-wave cutoff energy was chosen as 550 eV according to the convergence tests. The k-points set of 16x16x1 was chosen as optimal for all unit cell calculations and set of 4x4x1 k-points was used for calculation of best representative structures (4x4x1 size of supercell).

2 Results

Table S1: Calculated values of work function over whole concentration range of O, OH, and F, for Ti_2N, Ti_4N_3, Nb_2C, and Nb_4C_3

	Work function, eV					
Structure	Ti_2N	Ti_4N_3	Nb_2C	Nb_4C_3	Ti_2C	Ti_3C_2
$O_{0.25}OH_{0.75}$	1.6	1.6	1.5	1.7	1.7	1.7
$OH_{0.75}F_{0.25}$	1.7	1.7	1.8	1.5	1.4	1.3
$OH_{0.50}F_{0.50}$	1.9	1.9	1.9	2.0	2.2	2.1
$O_{0.25}OH_{0.50}F_{0.25}$	2.5	2.4	2.3	2.3	2.7	2.7
$O_{0.50}OH_{0.50}$	2.9	2.8	2.7	2.7	3.0	3.1
$OH_{0.25}F_{0.75}$	3.1	3.1	3.0	3.1	3.4	3.3
$O_{0.25}OH_{0.25}F_{0.50}$	3.7	3.6	3.4	3.5	3.9	3.9
$O_{0.50}OH_{0.25}F_{0.25}$	4.1	4.0	3.8	3.7	4.3	4.3
$O_{0.75}OH_{0.25}$	4.3	4.2	4.2	4.1	4.5	4.7
$O_{0.25}F_{0.75}$	5.2	5.1	4.8	4.9	5.3	5.3
$O_{0.50}F_{0.50}$	5.5	5.4	5.1	5.1	5.7	5.8
$O_{0.75}F_{0.25}$	5.8	5.7	5.5	5.4	5.9	6.1
$OH_{1.00}$	2.2	2.3	2.4	2.3	2.0	2.0
$O_{1.00}$	6.1	6.0	5.8	5.7	6.0	6.2
$F_{1.00}$	4.8	4.6	4.2	4.2	4.9	4.8

we evaluate the charges associated with each atom, using a Bader charge analysis. The averaged number of excess electrons, defined as the difference between the Bader charge and the number of valence electrons of the

corresponding species, is shown in the Fig. S4 as a function of O–OH concentration. The averaged number of electrons are associated with O and OH linearly depend on the amount of OH in the system [Fig. S4(a,c)], whereas the values are overall rather similar and we found no clear correlations with the maximum mixing energy or substrate and the number of atomic layers dependent features. On the other hand, charges of N and C atoms differ depending on the surrounding metallic species. In all Nb-based systems C does not change its charge significantly within a whole range of O-OH concentration, while the N and C atoms in Ti-based systems gain more electrons with an increase of OH content, additionally the carbon atoms in Ti-based systems take more electrons, than N atoms. Difference in the charge distributions might be one of the reason of different functional groups composition in Ti-based systems with carbon and nitrogen.

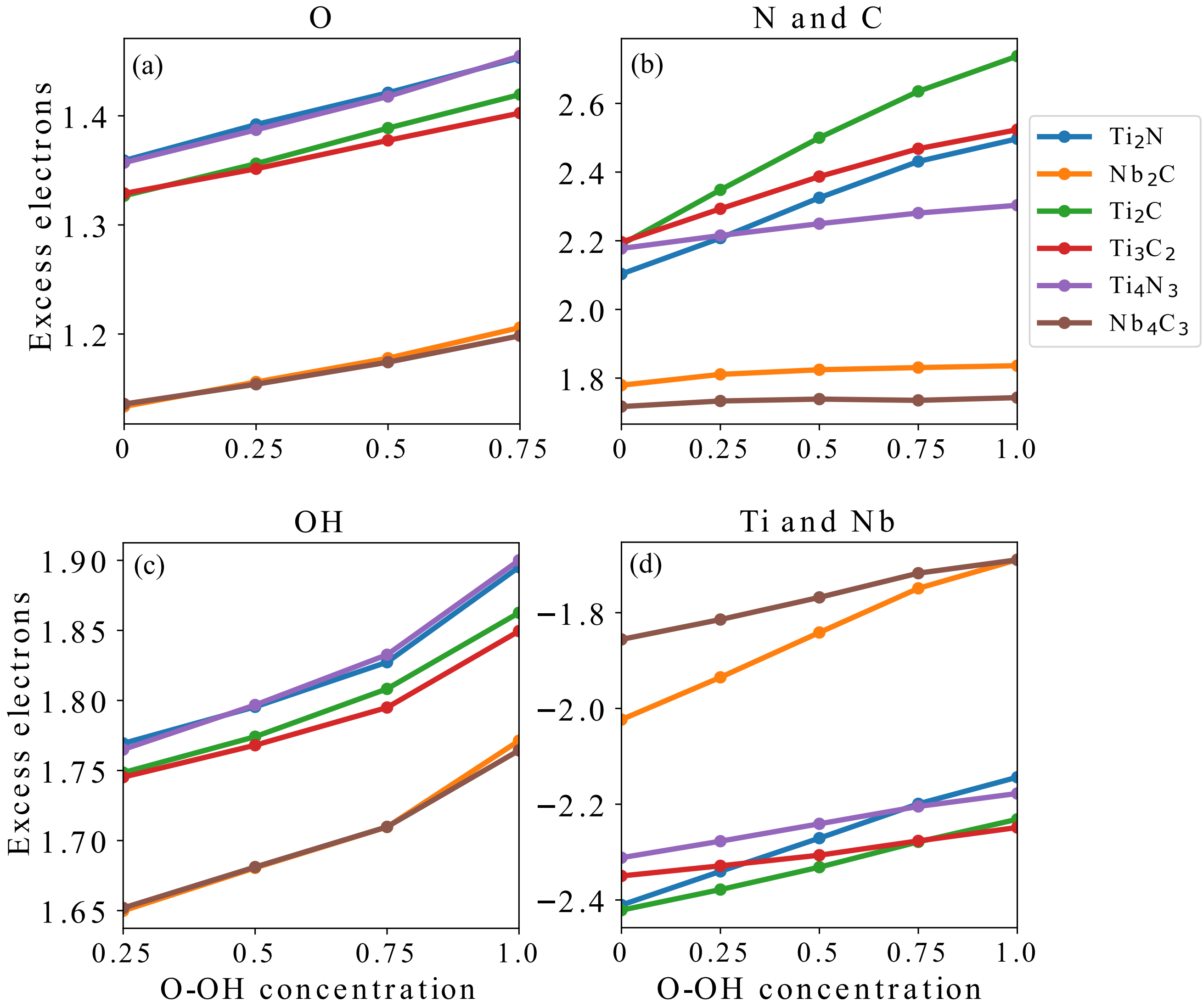

Figure S4: Averaged number of excess electrons associated with (a) O, (b) N, and C, (c) OH, and (d) Ti, and Nb atoms in Ti_2N, Ti_2C, Ti_3C_2, Ti_4N_3, Nb_2C, and Nb_4C_3 as a function of the O-OH composition.

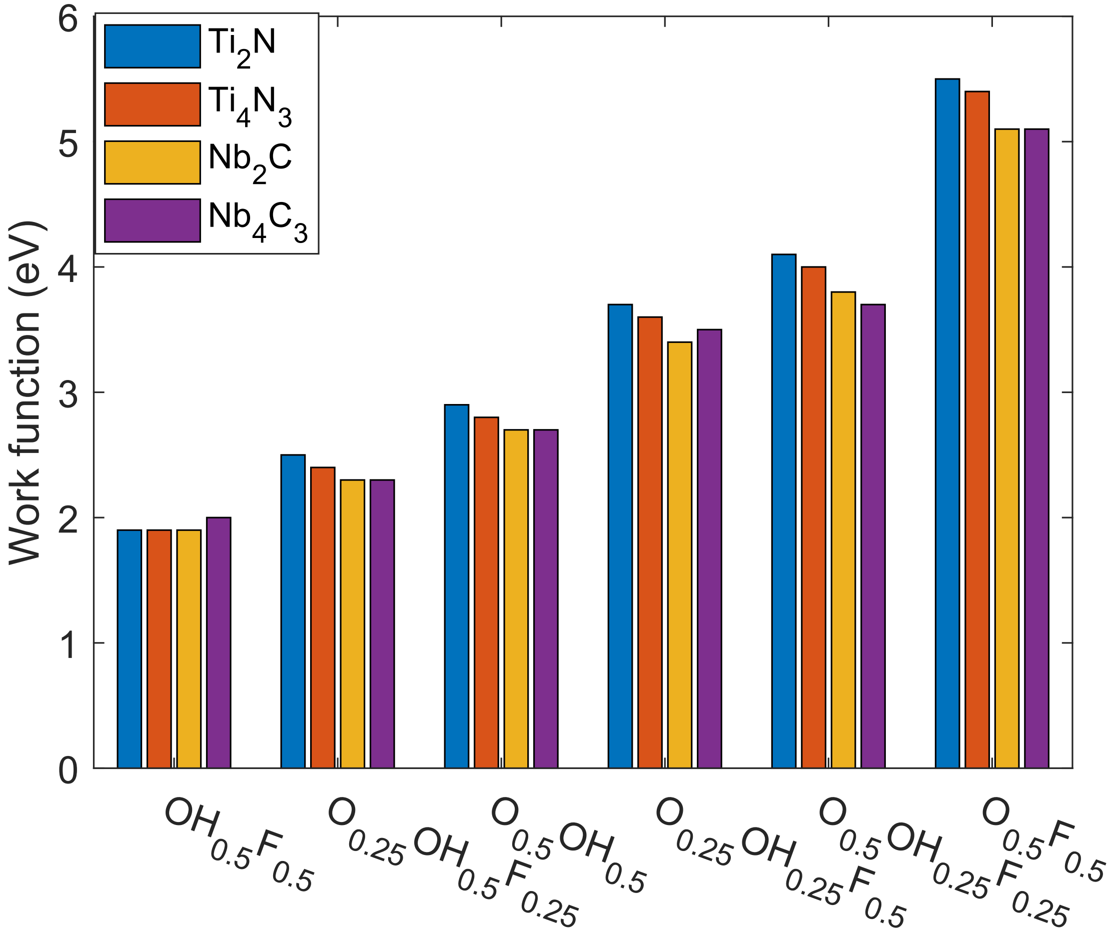

Figure S5: Work function (in eV) of binary and ternary mixtures on the surface of Ti_2N, Ti_4N_3, Nb_2C, Nb_4C_3.

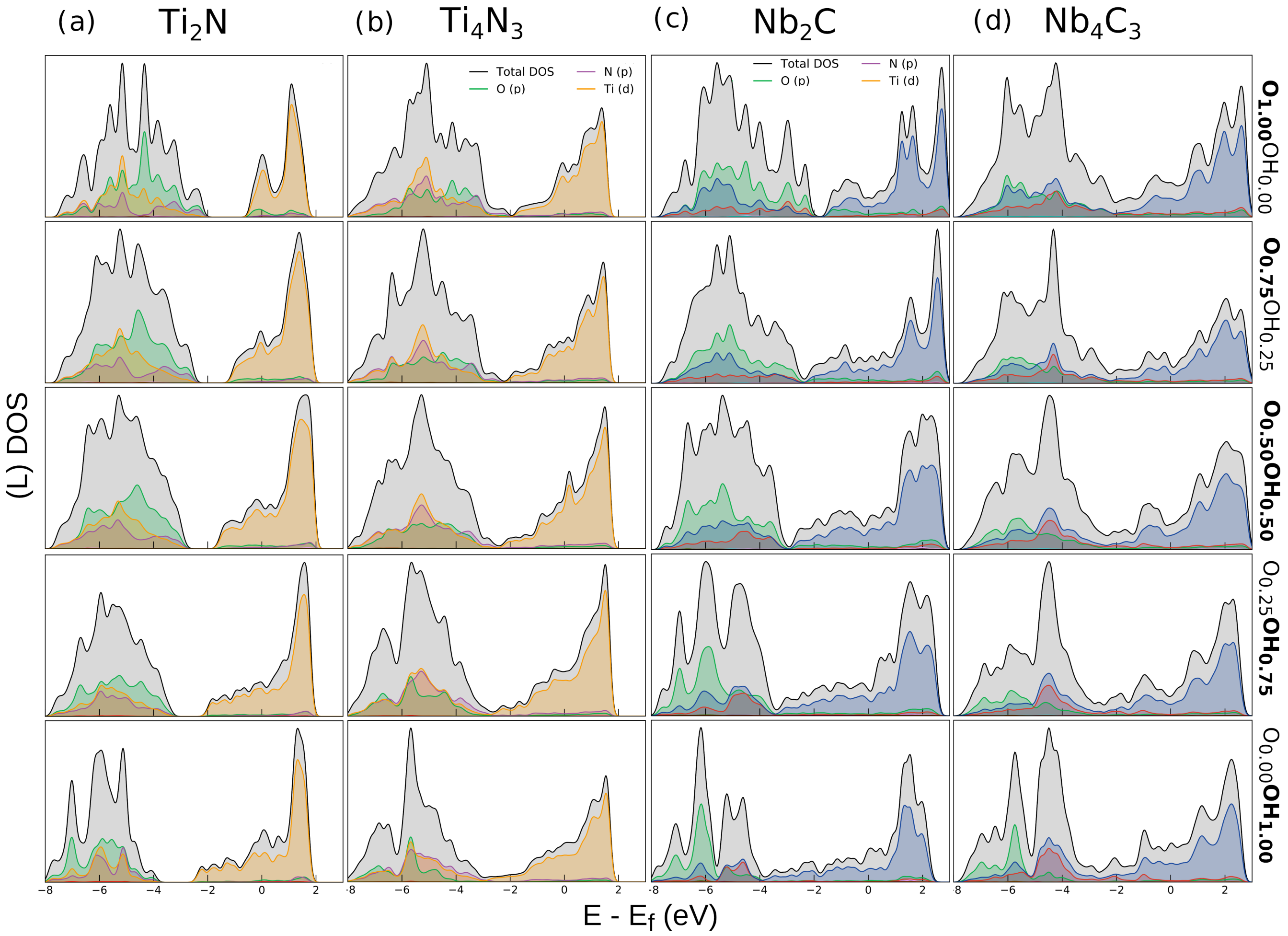

Figure S6: Atom-projected density of states from (a) Ti_2N, (b) Ti_4N_3, (c) Nb_2C, and (d) Nb_4C_3 SQoS.

References

[1] P. E. Blöchl. Projector augmented-wave method. *Phys. Rev. B*, 50:17953–17979, Dec 1994.

[2] Malcolm W. J. Chase. *NIST-JANAF Thermochemical Tables, 4th Edition*. American Institute of Physics, New York, 1998.

[3] Rina Ibragimova, Martti J. Puska, and Hannu-Pekka Komsa. ph-dependent distribution of functional groups on titanium-based mxenes. *ACS Nano*, 13(8):9171–9181, 2019.

[4] G. Kresse and J. Furthmüller. Efficient iterative schemes for ab initio total-energy calculations using a plane-wave basis set. *Phys. Rev. B*, 54:11169–11186, Oct 1996.

[5] Jian Liu, Maria V. Fernández-Serra, and Philip B. Allen. Special quasiordered structures: Role of short-range order in the semiconductor alloy $(GaN)_{1-x}(ZnO)_x$. *Phys. Rev. B*, 93:054207, Feb 2016.

[6] Yizhak Marcus. The thermodynamics of solvation of ions. part 2.-the enthalpy of hydration at 298.15 k. *J. Chem. Soc., Faraday Trans. 1*, 83:339–349, 1987.

[7] Yizhak Marcus. Thermodynamics of solvation of ions. part 5.-gibbs free energy of hydration at 298.15 k. *J. Chem. Soc., Faraday Trans.*, 87:2995–2999, 1991.

[8] John P. Perdew, Adrienn Ruzsinszky, Gábor I. Csonka, Oleg A. Vydrov, Gustavo E. Scuseria, Lucian A. Constantin, Xiaolan Zhou, and Kieron Burke. Restoring the density-gradient expansion for exchange in solids and surfaces. *Phys. Rev. Lett.*, 100:136406, Apr 2008.

[9] Mira Todorova and Jörg Neugebauer. Extending the concept of defect chemistry from semiconductor physics to electrochemistry. *Phys. Rev. Appl.*, 1:014001, Feb 2014.

[10] Alex Zunger, S.-H. Wei, L. G. Ferreira, and James E. Bernard. Special quasirandom structures. *Phys. Rev. Lett.*, 65:353–356, 1990.
